# Mammography at 40 saves lives: new evidence demands a global shift in breast cancer screening guidelines in Brazil

**DOI:** 10.1590/1806-9282.7112EDI

**Published:** 2026-01-09

**Authors:** Fernando Wladimir Silva Rivas, Rodrigo Gonçalves, Edmund Chada Baracat, José Maria Soares

**Affiliations:** 1 Universidade de São Paulo, Hospital das Clínicas, Faculdade de Medicina, Departamento de Obstetrícia e Ginecologia, Disciplina de Ginecologia – São Paulo (SP), Brazil.; 2 AstraZeneca R&D – Barcelona, Spain.

Breast cancer remains the leading cause of cancer-related mortality among women globally, with significant disparities in outcomes influenced by access to early detection, socioeconomic status, and educational attainment. In this context, the study by Rivas^1^ provides compelling evidence supporting the prognostic value of mammographic screening, particularly in reducing five-year mortality rates. The findings highlight that women whose breast cancer was detected via mammography before symptom onset had a 57% lower prevalence of advanced-stage disease and a 73% relative reduction in mortality compared to those diagnosed after symptoms appeared^1,2^. These results reinforce the life-saving potential of early detection, especially for women over 40 years of age^1^.

The debate over the optimal age to begin mammographic screening has long been contentious. While several guidelines advocate screening initiation at age 50, the findings from Rivas’ study offer important counterpoints^1^. The study's multivariate analyses revealed a 40% lower risk of death in women over 40 with nonmetastatic invasive disease when detected via mammography^1^. This challenges the notion that screening younger women yields minimal benefits, emphasizing instead the importance of early intervention in a population often diagnosed with aggressive tumor subtypes^3,4^.

Socioeconomic and racial disparities further complicate breast cancer outcomes. The study notes that nonwhite, unmarried, and lower-income women are less likely to undergo routine screening and more likely to experience higher mortality^1^. In Brazil, where access to healthcare is unevenly distributed, these disparities are stark^2^. Expanding screening programs to include women over 40 years of age could mitigate inequalities by catching cancers earlier in vulnerable populations, who often present with advanced-stage disease due to delayed diagnosis^5^.

Concerns regarding overdiagnosis and overtreatment have fueled criticism of early mammographic screening. However, Rivas’ data show that mammography-detected cancers are more likely to be treated with less invasive therapies, such as breast-conserving surgeries, and require fewer aggressive interventions like chemotherapy^1^. This approach not only contributes to improved survival outcomes but also preserves quality of life for patients. By advocating for individualized screening strategies rather than a one-size-fits-all model, the study addresses valid concerns while reinforcing the net benefit of early detection^6^.

The Brazilian context amplifies the urgency of this research. With low screening coverage and a high proportion of late-stage diagnoses, the country's breast cancer mortality rates remain alarmingly high. Rivas’ work aligns with global trends showing that organized screening programs reduce mortality, yet Brazil's opportunistic system leaves gaps^1^. Policymakers must prioritize structured screening initiatives, particularly for women over 40 years of age, to replicate the successes seen in high-income nations^7^.

The Riva's study also highlights the role of molecular subtypes in screening efficacy^1^. Luminal A tumors, more common in mammography-detected cases, have better prognoses, while aggressive subtypes like triple-negative cancers are often found in symptomatic women. This shift in subtype distribution with early detection highlights that the benefits of screening extend beyond improved survival; they also encompass a potential alteration in the biological profile of diagnosed cancers. By increasing the likelihood of detecting less aggressive tumors, screening may play a role in modifying the clinical course of the disease^2,4-6^.

Technological advancements and risk-based screening could further refine these efforts. Integrating tools like digital mammography and tomosynthesis, as suggested by recent studies, may improve detection rates and reduce false positives. Meanwhile, targeting high-risk groups—such as women with family histories or genetic predispositions—could optimize resource allocation without compromising public health gains^5-8^.

In conclusion, Rivas’ research provides compelling evidence for re-evaluating screening guidelines to include women over 40 years of age. The potential to reduce mortality, narrow disparities, and improve treatment outcomes is too significant to ignore. As breast cancer incidence rises globally, proactive screening policies must become a cornerstone of public health strategies, ensuring that early detection is accessible to all women, regardless of age or socioeconomic status. The time to act is now.

## Data Availability

The datasets generated and/or analyzed during the current study are available from the corresponding author upon reasonable request.
